# Albinisme oculocutané: iris en transillumination

**DOI:** 10.11604/pamj.2013.16.3.3154

**Published:** 2013-09-03

**Authors:** Hanan Handor, Mina Laghmari

**Affiliations:** 1 Université Mohammed V Souissi, Service d'Ophtalmologie A de l'hôpital des spécialités, Centre hospitalier universitaire, Rabat, Maroc

**Keywords:** Albinisme, iris, transillumination, albinism, iris, transillumination

## Image en médicine

L'albinisme regroupe un ensemble d'affections héréditaires liées à une anomalie de biosynthèse de la mélanine secondaire à un déficit variable en tyrosinase, enzyme impliquée dans la synthèse de ce pigment. La mélanine est élaborée dans des cellules spécialisées de la peau, des cheveux, de l’épithélium pigmenté de la rétine et au niveau de l'iris. L'examen à la lampe à fente d'un sujet albinos permet de mettre en évidence des signes caractéristiques de cette affection, notamment la transillumination irienne. Celle-ci est en rapport avec le déficit en mélanine du stroma et de l’épithélium postérieur de l'iris qui ne constituent plus une barrière pour la lumière réfléchie par la rétine. De ce fait les fibres zonulaires et l’équateur du cristallin peuvent être visibles.

**Figure 1:**
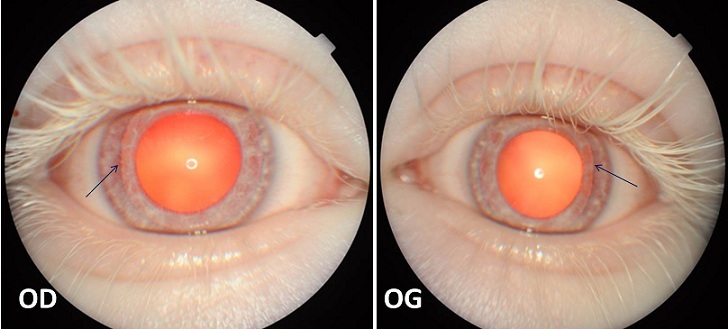
Aspect dépigmenté de l'iris avec une transillumination irienne permettant de voir l’équateur du cristallin en temporal au niveau des deux yeux (flèches bleues) au cours d'un albinisme oculocutané

